# Human Blood Index of *Anopheles arabiensis* in Ethiopia: A Systematic Review and Meta-Analysis

**DOI:** 10.1155/jotm/7891775

**Published:** 2025-08-31

**Authors:** Solomon Yeshanew, Fasil Adugna Kendie, Endalkachew Nibret, Getnet Atenafu

**Affiliations:** ^1^Department of Biology, College of Science, Bahir Dar University, Bahir Dar, Ethiopia; ^2^Department of Biology, Debre Markos University, Debre Markos, Ethiopia; ^3^Research/Development, Community & Industry Linkage Unit, Unity University, Addis Ababa, Ethiopia; ^4^Institute of Biotechnology, Bahir Dar University, Bahir Dar, Ethiopia; ^5^Sine-Hosaena Center, Bahir Dar University, Bahir Dar, Ethiopia

**Keywords:** *Anopheles arabiensis*, blood meal source, Ethiopia, human blood index, meta-analysis

## Abstract

**Background:** *Anopheles arabiensis*, the primary malaria vector in Ethiopia, exhibits diverse feeding behaviors influenced by geography, climate, and control strategies. Understanding its blood-feeding preference is crucial for devising effective interventions. This study aimed to conduct a systematic review and meta-analysis of existing evidence on *An. arabiensis* human blood index (HBI) in Ethiopia.

**Methods:** A comprehensive search of multiple electronic databases was conducted following the Preferred Reporting Items for Systematic Reviews and Meta-Analyses (PRISMA) guidelines. Study quality was assessed using criteria adopted from the Joanna Briggs Institute (JBI) appraisal checklist. Data were analyzed using Stata Version 17, employing a random-effects model to estimate the pooled HBI at 95% confidence interval (CI). Subgroup analysis and meta-regression were performed based on regions and mosquito collection methods. Heterogeneity was assessed using the *I*^2^ test.

**Results:** A total of 19 studies published from 1997 to 2023 were included, encompassing 12,794 blood-fed *An. arabiensis*. The meta-analysis revealed a pooled HBI of 37.18% (95% CI: 21.26–44.28). Subgroup analysis showed regional variation, with the highest HBI reported in Mixed Region 3 (covering Afar, Oromia, and the Amhara Regional States) at 64.02% (95% CI: 61.78–66.25), and the lowest in the Amhara Regional State at 7.53% (95% CI: −1.58–16.65). Temporal analysis indicated fluctuations over time, with the highest HBI reported in 2014 (70.62%, 95% CI: 68.72–72.46) and the lowest in 2021 (0%, 95% CI: 0.00–1.30).

**Conclusion:** The present study found that *An. arabiensis* in Ethiopia exhibits a moderate preference for human blood, with a pooled HBI of 37.18%. However, significant variation exists across regions and over time. Continuous surveillance and further research are needed to explore the underlying factors influencing HBI and to guide evidence-based malaria prevention and control strategies.

## 1. Background

Ethiopia, a sub-Saharan African country, bears a substantial malaria burden, with approximately 68% of its landmass vulnerable to transmission and about 60% of the population at risk [1, 2]. Malaria remains a major public health challenge, accounting for over 1.2 million outpatient visits annually [1, 3, 4]. Control efforts primarily rely on chemical interventions, including early detection and treatment of infection, as well as the use of insecticides for vector control. However, the effectiveness of these strategies is increasingly threatened by the emergence of multidrug-resistant malaria parasites and widespread insecticide resistance among the principal vectors [5, 6].

*Plasmodium falciparum* and *Plasmodium vivax* are the two dominant malaria parasites in Ethiopia, and *An. arabiensis*, the principal vector, plays a key role in transmitting both [1, 5, 7]. The abundance of this mosquito species typically peaks following the main rainy season (September–November) across most malaria-endemic areas [8, 9]. In contrast, the western and southwestern regions typically receive more consistent rainfall, supporting year-round mosquito breeding and perennial malaria transmission [5].

*An. arabiensis* is highly adaptable, tolerating a wide range of climatic and ecological conditions across diverse geographical settings such as rugged mountains, flat-topped plateaus, deep gorges and river valleys, and fertile plains with the highest population densities often recorded in dry, sparsely wooded, and urban areas [7, 10–12]. Consequently, its blood-feeding patterns, host preferences, and resting and biting behaviors vary significantly across different ecological zones [9, 11, 12]. Among these factors, blood meal preference is particularly critical in influencing malaria transmission dynamics and the broader epidemiology of the disease [13].

Understanding the feeding behavior of *An. arabiensis*, particularly its preference for human blood, is essential for designing and implementing effective malaria control strategies [14, 15]. The human blood index (HBI) of *An. arabiensis*, defined as the proportion of blood meals taken from humans relative to all blood meals taken, is a key indicator of the mosquito's vectorial capacity and its role in malaria transmission dynamics [16]. The HBI of *An. arabiensis* varies across regions and seasons, influenced by factors such as climate, land use, human population density, and vector control interventions [12, 15]. Therefore, gaining comprehensive understanding of the HBI of *An. arabiensis* in the Ethiopian context is vital for guiding targeted and evidence-based vector control efforts.

This systematic review and meta-analysis aimed to assess the existing evidence on the HBI of *An. arabiensis* in Ethiopia. By systematically identifying, appraising, and synthesizing data from original studies, the review sought to provide a comprehensive overview of the extent to which *An. arabiensis* fed on humans across various regions and ecological settings in the country. Through quantitative meta-analysis, the study further explored patterns, trends, and potential determinants of human blood feeding. The findings are expected to inform and strengthen malaria control strategies by identifying high-risk areas and populations where *An. arabiensis* demonstrates a strong preference for human hosts.

## 2. Methods

### 2.1. Search Design and Strategy

This systematic review adhered to the Preferred Reporting Items for Systematic Reviews and Meta-Analyses (PRISMA) guidelines (Table S1). A comprehensive search was conducted across electronic databases, including Google Scholar, PubMed, ScienceDirect, and African Journals Online, to identify relevant articles. Additional articles were located through manual searches on Google and screening of reference lists. The search was completed on December 31, 2023, with no restrictions on publication year. Search terms were selected based on Medical Subject Heading (MeSH) terms. Keywords such as “*Anopheles* mosquito,” “malaria vectors,” “blood meal preference/blood meal analysis/blood meal source/blood meal origin,” “Ethiopia,” “*Anopheles arabiensis*,” and “*Anopheles arabiensis* feeding behavior” were included either individually or in combination with Boolean operators (Table S2). All retrieved articles were exported to EndNote Version X8 (Thomson Reuters, USA) for duplicate removal.

### 2.2. Article Eligibility Criteria

#### 2.2.1. Inclusion Criteria

The review focused on studies examining the HBI of *An. arabiensis* in Ethiopia. Original studies conducted in Ethiopia that reported blood meal analysis using techniques such as PCR, ELISA, or precipitin tests were included. Studies were eligible if they provided data on HBI, adult mosquito collection methods (e.g., CDC-LTs, PSC, APS, and manual aspiration), indoor and outdoor collection settings, and the total number of blood-fed *An. arabiensis* captured.

#### 2.2.2. Exclusion Criteria

Studies were excluded if they did not report the origin of human blood meals, failed to provide the total number of blood-fed *An. arabiensis* mosquitoes captured, or utilized host-biased trapping methods. Experimental studies conducted in controlled laboratory settings (in vitro studies) were also excluded. Additionally, articles without full text were excluded after at least three unsuccessful attempts to contact the primary author via email. Non-English publications, books, and review articles were also excluded from the review.

### 2.3. Article Selection and Quality Assessment

Two authors (Solomon Yeshanew and Fasil Adugna Kendie) independently screened the titles and abstracts to assess eligibility. Full-text versions of potentially relevant studies were then obtained and evaluated against the predefined inclusion criteria. The quality of the included articles was assessed independently by the same authors using tools adopted from the Joanna Briggs Institute (JBI) appraisal checklist [17]. Each study was evaluated based on nine criteria with a cutoff point of 50% for inclusion. Any disagreements regarding article inclusion or quality assessment were resolved through discussion with the third (Endalkachew Nibret) and fourth (Getnet Atenafu) authors.

### 2.4. Data Extraction and Analysis

#### 2.4.1. Data Extraction

A standardized data extraction form was developed using Microsoft Excel to collect relevant information from the full-text articles. The form included fields such as first author's name, year of publication, study site (region and geographic location), mosquito collection settings (indoor, outdoor, or both), collection methods (CDC light traps [CDC-LTs], PSC, APS, etc.), HBI, bovine blood index (BBI), mixed blood index (MBI), unidentified blood index (UBI), and blood meal analysis techniques used (PCR, ELISA, and precipitin) (Table S3). The extracted data from both authors (Solomon Yeshanew and Fasil Adugna Kendie) were systematically reviewed for consistency. The other two authors (Endalkachew Nibret and Getnet Atenafu) then conducted a final review and reached an agreement on the data abstraction.

#### 2.4.2. Data Analysis

Data were analyzed using Stata software (Version 17, StataCorp, College Station, TX) employing the “metan” command. A forest plot was generated to estimate the pooled prevalence of HBI with a 95% confidence interval (CI). Heterogeneity among studies was assessed using the inverse variance index (*I*^2^) statistics, with values categorized as low (< 25%), moderate (25%–50%), and high (> 50%) heterogeneity [18]. Due to substantial heterogeneity in HBI estimates across studies, a random-effect model was applied. Subgroup and meta-regression analysis were conducted to explore potential sources of heterogeneity. Publication bias was assessed qualitatively through funnel plot symmetry and quantitatively using Egger's regression test.

## 3. Results

### 3.1. Study Selection

**Figure 1:**
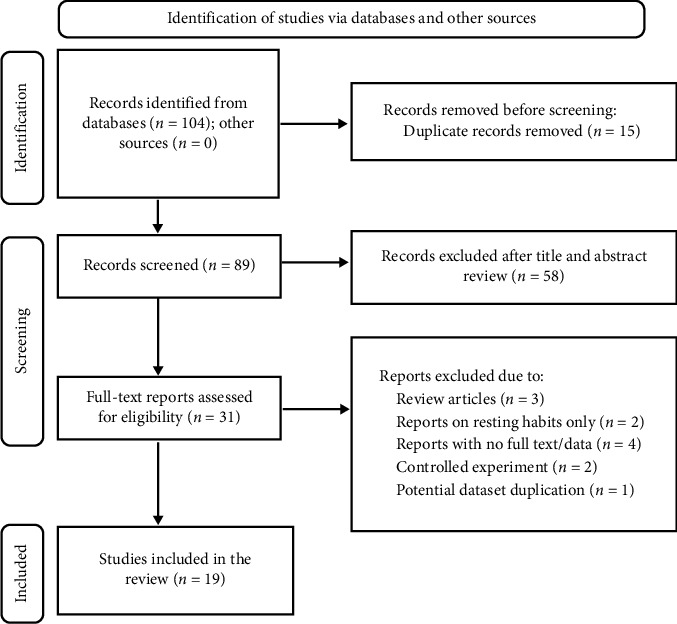
The PRISMA (2020) flow diagram illustrating the study selection processes.

The initial search identified 104 potentially relevant studies. After removing 15 duplicates, 89 were screened based on their titles and abstracts, leading to the exclusion of 58 articles. The remaining 31 articles underwent full-text screening, from which 19 met the eligibility criteria and were included in the meta-analysis (Table 1). The study selection process adhered to the PRISMA flow diagram (Figure 1).

### 3.2. Characteristics of Included Articles

**Figure 2:**
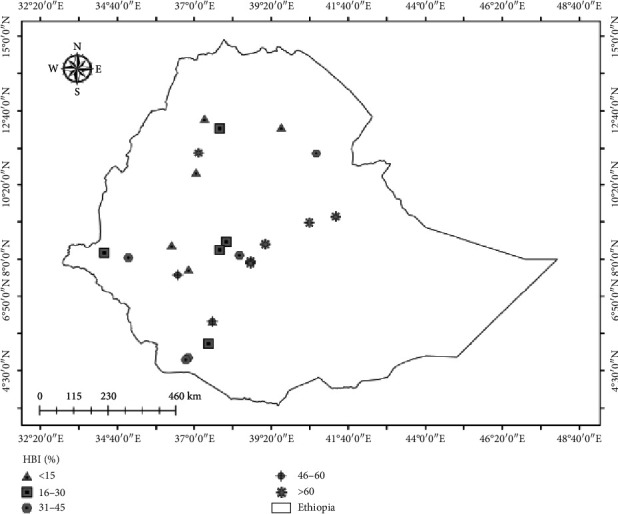
Geographic distribution of study sites and estimated HBI of *An. arabiensis* in Ethiopia (source: study sites depicted on the map are extracted from included studies in this systematic review).

A total of 19 articles were included, encompassing 12,794 blood-fed *An. arabiensis* mosquitoes. The number of mosquitoes per study ranged from 7 to 2335. These studies, published between 1997 and 2023, were conducted across five regional states, with the highest proportion (31.6%) originating from the SNNP Regional State (Figure 2). Over 10 different mosquito collection methods were employed across the studies. The overall proportion of HBI was 37.18%, followed by BBI (28.61%). Additionally, 15.40% of mosquitoes had mixed human and bovine blood meals, while the remaining had unknown blood meal sources (Table 1).

### 3.3. Quality Assessment

All studies included in this review were evaluated using the nine-item JBI quality assessment tool for studies on human blood meal sources. Each item was scored as “yes” = 1 and “no” or “unclear” or “not applicable” = 0. Of the 19 articles, 11 scored seven or more points, indicating good methodological quality (Table S4). The overall quality score was 77.78%, well above the cutoff point of 50%, suggesting a low risk of bias among the included studies (Table 1).

### 3.4. Proportions of Human Blood Meal in *An. arabiensis*

**Figure 3:**
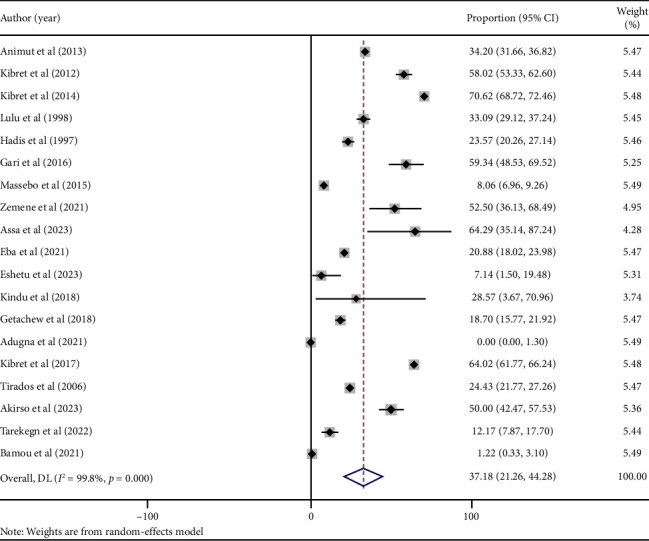
Forest plot showing the pooled estimate of HBI among blood-fed *An. arabiensis* mosquitoes collected in Ethiopia (the darker vertical hatched line at 0 represents the null effect; the lighter hatched lines indicate the 95% CI; squares represent point estimates for individual studies; the diamond represents the overall pooled estimate).

The pooled proportion of human blood meals in *An. arabiensis* across studies conducted in Ethiopia was 37.18% (95% CI: 21.26–44.28). The lowest reported proportion was 0% (95% CI: 0.00–1.30), recorded in Bure district, Amhara Regional State [32], while the highest was 70.62% (95% CI: 68.72–72.46), observed in Ziway, Oromia Regional State [20]. Substantial heterogeneity was noted among studies (*I*^2^ = 99.8%, *p* < 0.001) (Figure 3).

### 3.5. Subgroup Analysis by Regional States and Mosquito Collection Methods

**Figure 4:**
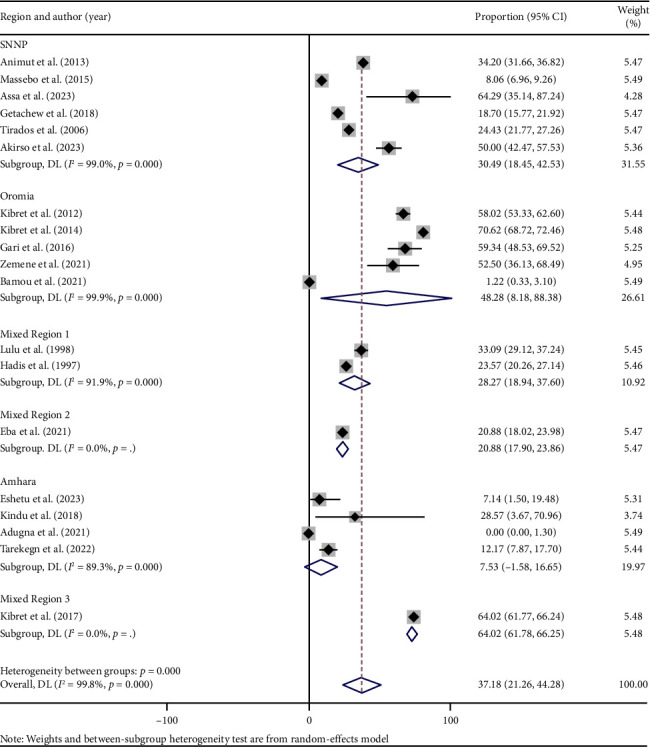
Forest plot showing the pooled proportion of human blood meals among blood-fed *An. arabiensis* mosquitoes by regional states in Ethiopia.

**Figure 5:**
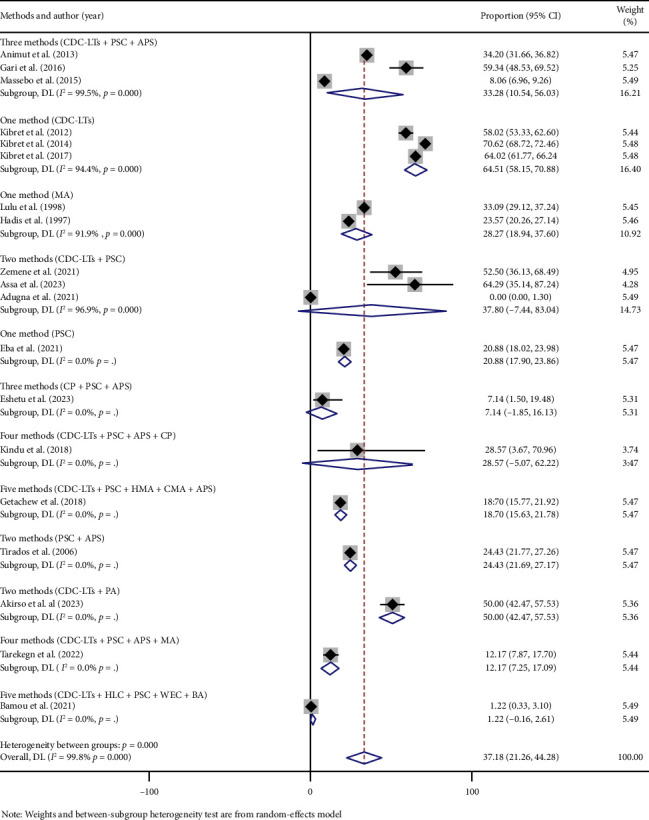
Forest plot showing the pooled proportion of human blood meals among blood-fed *An. arabiensis* mosquitoes by collection methods in Ethiopia.

Subgroup analysis indicated variations in the human blood meal proportion of *An. arabiensis* across regional states and mosquito collection methods. The highest proportion was recorded in Mixed Region 3, at 64.02% (95% CI: 61.78–66.25), followed by Oromia Regional State with 48.28% (95% CI: 8.18–88.38). The lowest proportion was observed in Amhara Regional State at 7.53% (95% CI: −1.58–16.65) (Figure 4). Regarding mosquito collection method, the highest pooled human blood meal proportion was reported in studies using only CDC-LTs, at 64.51% (95% CI: 56.15–70.88) [20, 21, 33]. This was followed by studies using a combination of CDC-LTs and PA, reporting 50.0% (95% CI: 42.47–57.53) [35]. In contrast, the lowest proportion, 0%, was documented in a study using both CDC-LTs and PSC [32] (Figure 5).

### 3.6. Publication Bias Across Studies

**Figure 6:**
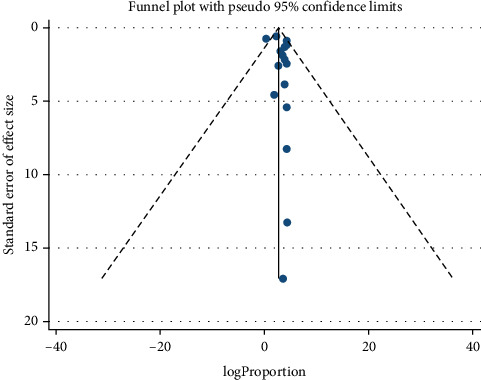
Funnel plot assessing the presence of publication bias among the included studies.

The risk of publication bias was evaluated using the nine-item JBI appraisal checklist [17], with scores ranging from 0 to 9, where higher scores indicate better quality. Overall, the included studies were deemed to be of good quality (Table 1). The publication bias was further assessed both subjectively using funnel plot types and objectively using Egger's regression test. The funnel plot showed asymmetry, suggesting possible publication bias (Figure 6). However, Egger's regression test results (*p* = 0.134) indicated no statistically significant evidence of publication bias across the studies (Table 2).

### 3.7. Trend Patterns of HBI Over Time

**Figure 7:**
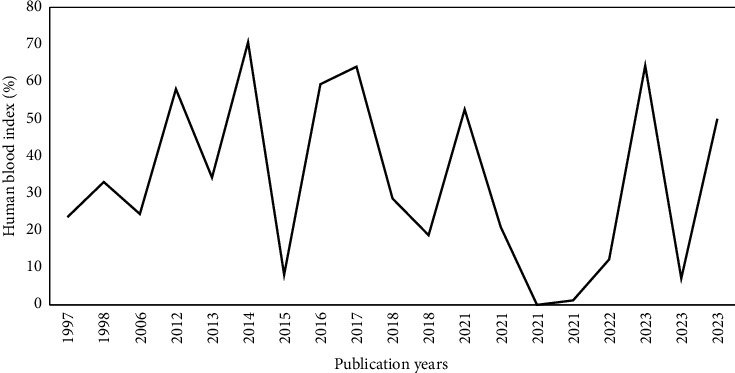
Trend patterns of HBI of *An. arabiensis* in Ethiopia from 1997 to 2023.

The analysis showed that the proportion of human blood meals in *An. arabiensis* did not follow a consistent trend between 1997 and 2023. Instead, fluctuations of sharp increases and decreases were observed over the 27-year period. The highest proportion was reported in 2014 (70.6%) [20], followed by 2023 (64.3%) [27], and 2016 (59.3%) [24]. In contrast, the lowest HBI was reported in 2021, with no human blood meal detected [32] (Figure 7).

## 4. Discussion

This systematic review and meta-analysis revealed that *An. arabiensis* in Ethiopia has an overall HBI of 37.18%. When accounting for mosquitoes that fed on both humans and bovines (MBI = 15.40%), the combined proportion of mosquitoes that included human blood in their meals rises to 52.58%. This finding highlights the significant role of humans as a primary blood meal source for *An. arabiensis*, indicating its potential contribution to malaria transmission in Ethiopia. However, the observed proportion varied widely across studies, ranging from 0% [32] to 70.62% [20], reflecting the presence of substantial heterogeneity across different geographic locations and ecological settings. These variations suggest complex feeding behaviors and underline the importance of local context in understanding mosquito–host interactions and transmission dynamics.

In rural Ethiopia, it is common for humans and livestock to share the same living spaces or compounds, creating opportunities for *An. arabiensis* to alternate between human and animal hosts. This zoophilic-anthropophilic flexibility contributes to the mosquito's adaptability and persistence in diverse environments. To effectively reduce malaria transmission, integrated vector management strategies are essential. The combination of long-lasting insecticidal nets (LLINs) with the systematic use of insecticide-treated livestock has shown promise in controlling mosquito populations and interrupting malaria transmission cycles [14]. Additionally, the use of endectocides such as ivermectin in livestock offers a complementary approach by killing mosquitoes that feed on treated animals and reducing malaria parasite development within vectors, further enhancing control efforts [38]. Supporting this, Wang et al. identified HBI as the most influential bionomic parameter in designing effective vector control strategies for other mosquito species [39].

The observed heterogeneity across different locations and settings underscores the multifactorial nature of the mosquito's feeding preferences. These variations may be attributed to ecological differences, human population densities, mosquito behaviors, and the use of personal protective measures such as bed nets [12, 15]. Additionally, discrepancies in study design, mosquito sampling techniques, and blood meal analysis methods may also contribute to the variability observed across studies [40, 41], making it difficult to draw definitive conclusions from the pooled data.

For instance, the lowest and highest HBI values were reported in the Bure district of the Amhara Regional State (0%) and Ziway areas of the Oromia Regional State (70.62%), respectively. Several factors may explain the low HBI in Bure, including widespread use of bed nets, the presence of numerous domestic animals as alternative hosts, a moderate climate (relatively cooler temperature or “Woyina Dega” agro-ecology), and the absence of suitable mosquito breeding habitat during the dry season [32]. In contrast, the high HBI reported in Ziway may be linked to the presence of permanent water bodies (e.g., Lake Ziway), elevated temperature, and extensive irrigation practices, all of which create favorable conditions for mosquito breeding and increase human–vector contact [20].

Similarly, subgroup analysis based on regional states and mosquito collection methods showed significant variability in HBI across different geographical area and mosquito collection tools. Regions such as Mixed Region 3 [33] and Oromia Regional State [20] reported notably higher HBI proportions compared to others. This elevated HBI may be linked to the presence of lakes and irrigation systems, which create conducive breeding conditions to sustain *An. arabiensis* year-round. Furthermore, HBI estimates also varied depending on mosquito collection methods employed, with CDC-LTs consistently yielding the highest values [20, 21, 33]. These findings thus emphasize the importance of employing standardized methodologies in research and surveillance efforts to ensure consistency and comparability of results across different studies and regions.

Temporal fluctuations of HBI further highlight the dynamic nature of *An. arabiensis* feeding behaviors over time. The trend shows sharp increases and decreases across different study periods, likely influenced by factors such as seasonal variations, climate change, and the implementation of vector control interventions, all of which can affect the availability and attractiveness of human hosts [42, 43]. The highest HBI was recorded in 2014 [20], followed by that in 2023 [27], while the lowest was reported in 2021 [32], signifying the mosquito's adaptive elasticity in selecting blood meal sources over time. These fluctuations underscore the importance of continuous surveillance to monitor shifts in mosquito feeding patterns and guide responsive vector control strategies. However, the interpretation of these temporal trends is limited by the scarcity of long-term data and the lack of detailed environmental and socioeconomic context in the included studies.

The assessment of publication bias using both funnel plots and Egger's regression test yielded mixed results. While the funnel plot asymmetry suggests the presence of bias, Egger's regression tests, on the other hand, provide a statistical approach that did not detect bias across studies. This inconsistency may stem from several factors. One possibility is publication bias inherent to systematic reviews that rely solely on published literature, where studies with significant or positive findings are more likely to be published, potentially inflating effect estimates. The other reason might be due to the small sample size, small-study effect, and variability in study characteristics which could also contribute to the observed discrepancies. Although statistical approaches indicated no significant publication bias, these results should be interpreted with caution, as the possibility of underlying bias cannot be entirely excluded.

## 5. Conclusion

The pooled HBI of *An. arabiensis* in Ethiopia was 37.18%, with significant variation observed across geographic regions, study design, and period. The findings provide valuable information for malaria control efforts by highlighting regional disparities, methodological influences, and temporal shifts in mosquito feeding behavior. Such evidence can support the design of more targeted and effective vector control strategies. However, further research is essential to better understand drivers of these variations across different ecological and sociodemographic settings. This will be critical for developing context-specific and evidence-based interventions to enhance malaria prevention and control in Ethiopia.

## Figures and Tables

**Table 1:** Characteristics of the included studies on the blood meal origin of *An. arabiensis* in Ethiopia.

Author name	Publication year	Region	Collection methods	Total blood-fed mosquito	HBF	BBF	MBF	UBF	OQS
Animut et al. [19]	2013	SNNP	CDC-LTs, PSC, APS	1336	457	393	167	319	9
Kibret et al. [20]	2014	Oromia	CDC-LTs	2335	1649	476	133	77	7
Kibret et al. [21]	2012	Oromia	CDC-LTs	455	264	70	50	71	6
Lulu et al. [22]	1998	Mixed Region 1	MA	538	178	138	31	191	5
Hadis et al. [23]	1997	Mixed Region 1	MA	611	144	170	11	286	5
Gari et al. [24]	2016	Oromia	CDC-LTs, PSC, APS	91	54	26	9	2	7
Massebo et al. [25]	2015	SNNP	CDC-LTs, PSC, APS	2234	180	745	807	502	9
Zemene et al. [26]	2021	Oromia	CDC-LTs, PSC	40	21	11	6	2	5
Assa et al. [27]	2023	SNNP	CDC-LTs, PSC	14	9	2	0	3	5
Eba et al. [28]	2021	Mixed Region 2	PSC	747	156	266	81	244	7
Eshetu et al. [29]	2023	Amhara	CP, PSC, APS	42	3	14	2	23	6
Kindu et al. [30]	2018	Amhara	CDC-LTs, PSC, APS, CP	7	2	2	0	3	6
Getachew et al. [31]	2018	SNNP	CDC-LTs, PSC, APS, HMA, CMA	647	121	350	7	169	9
Adugna et al. [32]	2021	Amhara	CDC-LTs, PSC	209	0	6	191	12	9
Kibret et al. [33]	2017	Mixed Region 3	CDC-LTs	1818	1164	427	169	58	8
Tirados et al. [34]	2006	SNNP	CDC-LTs, PSC	974	238	354	278	104	8
Akirso et al. [35]	2023	SNNP	CDC-LTs, PA	180	90	1	0	89	6
Tarekegn et al. [36]	2022	Amhara	CDC-LTs, PSC, APS, MA	189	23	68	6	92	9
Bamou et al. [37]	2021	Oromia	CDC-LTs, HLC, PSC, WEC, BA	327	4	141	23	159	7

*Note:* Mixed Region 1: SNNP, Gambella, and Afar; Mixed Region 2: Oromia and SNNP; Mixed Region 3: Afar, Oromia, and Amhara.

Abbreviations: APS = artificial pit shelter, BA = backpack aspirator, BBF = bovine blood-fed, CDC-LTs = Centers for Disease Control and Prevention light traps, CMA = cattle-mediated aspirator, CP = clay pot, HBF = human blood-fed, HLC = human landing catch, HMA = human-mediated aspirator, MA = mouth aspirator, MBF = mixed blood-fed, OQS = overall quality score, PA = Prokopack aspirator, PSC = permethrin spray catch, UBF = unknown blood-fed, and WEC = window exit trap.

**Table 2:** Results of Egger's meta-regression test assessing the absence of small-study effects.

Egger's test for small-study effects: Regress standard normal deviate of intervention effect estimate against its standard error						
Number of studies = 19				Root MSE = 1.007		
Std_Eff	Coef.	Std. err	*t*	*p* > |*t*|	(95% CI)	
Slope	1.784973	0.5367409	3.33	0.004	0.6471331	2.922813
Bias	0.6247631	0.3954358	1.58	0.134	−0.2135234	1.46305

*Note:* Test of H0: no small-study effects, *p*=0.134.

## Data Availability

All data analyzed during this study are included in this manuscript/supporting information.

## Nomenclature

APS: Artificial pit shelter
BBI: Bovine blood index
CDC-LTs: Centers for Disease Control and Prevention light traps
CI: Confidence interval
ELISA: Enzyme-linked immunosorbent assay
FMOH: Federal Ministry of Health, Ethiopia
HBI: Human blood index
JBI: Joanna Briggs Institute
LLINs: Long-lasting insecticidal nets
MBI: Mixed blood index
MeSH: Medical Subject Heading
OQS: Overall quality score
PCR: Polymerase chain reaction
PMI: President's Malaria Initiative
PRISMA: Preferred Reporting Items for Systematic Reviews and Meta-Analyses
PSC: Pyrethrum spray catch
SNNP: Southern Nations, Nationalities, and Peoples
UBI: Unknown blood index
WHO: World Health Organization

## Supporting Information

Supporting Information 1 Table S1: PRISMA 2020 checklist completed for “Human blood index of *Anopheles arabiensis* in Ethiopia: a systematic review and meta-analysis.”

Supporting Information 2 Table S2: database-specific search strategies and dates.

Supporting Information 3 Table S3: data of all eligible studies included in the review.

Supporting Information 4 Table S4: quality assessment chart of the included articles.
