# Supplementary material for: Human Blood Index of Anopheles arabiensis in Ethiopia: A Systematic Review and Meta-Analysis
Source: J Trop Med. 2025 Aug 31;2025:7891775. doi: 10.1155/jotm/7891775 (PMC12414622; doi:10.1155/jotm/7891775)
Supplement: Supporting Information 1 — Table S1: PRISMA 2020 checklist completed for “Human blood index of Anopheles arabiensis in Ethiopia: a systematic review and meta-analysis.” [file 7891775.f1.docx]

**Table S1:** PRISMA 2020 Checklist-Completed for 'Human blood index of *Anopheles arabiensis* in Ethiopia: a systematic review and meta-analysis'

| **Section/Topic** | **Checklist Item** | **Reported on page #** |
| --- | --- | --- |
| Title | Identify the report as a systematic review and/or meta-analysis. | Page 1, Title page |
| Abstract | Structured summary of background, objectives, data sources, eligibility criteria, methods, results, and conclusions. | Page 2-3, Abstract |
| Rationale | Describe the rationale for the review in the context of what is already known. | Page 3-4, Background |
| Objectives | Provide an explicit statement of the objective(s) or question(s) being addressed. | Page 4, Background last paragraph |
| Eligibility criteria | Specify the inclusion and exclusion criteria for the review and how studies were grouped. | Page 5, 'Article eligibility criteria' |
| Information sources | Specify all databases, registers, websites, organisations, reference lists, and other sources searched or consulted to identify studies. Specify the date last searched. | Page 4-5, 'Search design and strategy' |
| Search strategy | Present the full search strategies for all databases, including any filters and limits used. | Page 4-5, 'Search design and strategy' |
| Selection process | Specify the methods used to decide whether a study met the inclusion criteria and how many reviewers screened each record and each report retrieved. | Page 6, 'Article selection and quality assessment' |
| Data collection process | Specify methods used to collect data from reports, how many reviewers collected data, and any processes for obtaining or confirming data. | Page 6-7, 'Data extraction and analysis' |
| Data items | List and define all outcomes for which data were sought. | Page 6-7, 'Data extraction and analysis' |
| Risk of bias assessment | Describe any methods used to assess risk of bias in the included studies. | Page 6-7, 'Article selection and quality assessment'' |
| Effect measures | Specify for each outcome the effect measure(s) used in the synthesis or presentation of results. | Page 6-7, Data analysis (pooled prevalence with 95% CI) |
| Synthesis methods | Describe the methods of handling data and combining results of studies. | Page 6-7, 'Data analysis' |
| Study selection results | Provide number of studies screened, assessed for eligibility, and included in the review, with reasons for exclusions, ideally using a flow diagram. | Page 7, 'Study selection' and Figure 1 |
| Study characteristics | Present characteristics for each study and provide the citations. | Page 8-9, Table 1 |
| Risk of bias in studies | Present data on risk of bias of each study and, if available, any outcome-level assessment. | Page 8, 'Quality assessment' |
| Results of individual studies | For all outcomes, present summary statistics for each study. | Page8- 9, Table 1 |
| Results of syntheses | Present the results of all statistical syntheses conducted. | Page 9-15, Figures 3-5 |
| Reporting bias assessment | Present assessments of risk of bias due to missing results (arising from reporting biases) for each synthesis assessed. | Page 13-14, 'Publication bias across studies' and Figure 6 |
| Certainty assessment | Present assessments of certainty (or confidence) in the body of evidence for each outcome assessed. | Not explicitly stated |
| Discussion | Summarise the main findings, discuss limitations, and implications for practice and research. | Page 15-18, 'Discussion' |
| Competing interests | Declare any competing interests of review authors. | Page 19, 'Competing interests' |
| Availability of data, and other materials | data used for all analyses; analytic code; any other materials used in the review | Page 19, 'Availability of data' |
| Funding | Describe sources of funding and other support; role of funders. | Page 19, 'Funding' |
