# Supplementary material for: Human Blood Index of Anopheles arabiensis in Ethiopia: A Systematic Review and Meta-Analysis
Source: J Trop Med. 2025 Aug 31;2025:7891775. doi: 10.1155/jotm/7891775 (PMC12414622; doi:10.1155/jotm/7891775)
Supplement: Supporting Information 2 — Table S2: database-specific search strategies and dates. [file 7891775.f2.docx]

**Table S2:** Database-Specific Search Strategies and Dates

| **Database** | **Search Terms Used** | **Boolean Operators Used** | **Date Searched** |
| --- | --- | --- | --- |
| PubMed | ("Anopheles mosquito" OR "Anopheles arabiensis") AND ("malaria vectors") AND ("blood meal preference" OR "blood meal analysis" OR "blood meal source") AND "Ethiopia" | AND, OR | 15 – 20 November 2023 |
| ScienceDirect | "Anopheles arabiensis" AND ("feeding behavior" OR "blood meal origin") AND "Ethiopia" | AND, OR | 23 - 27 November 2023 |
| Google Scholar | "Anopheles mosquito" OR "malaria vectors" OR "Anopheles arabiensis feeding behavior" OR "blood meal source" AND "Ethiopia" | OR, AND | 05 - 12 December 2023 |
| African Journals Online (AJOL) | ("Anopheles mosquito" AND "malaria vectors") OR ("blood meal analysis") OR ("Anopheles arabiensis") AND "Ethiopia" | AND, OR | 22 – 31 December January 2023 |
