# Supplementary material for: Human Blood Index of Anopheles arabiensis in Ethiopia: A Systematic Review and Meta-Analysis
Source: J Trop Med. 2025 Aug 31;2025:7891775. doi: 10.1155/jotm/7891775 (PMC12414622; doi:10.1155/jotm/7891775)
Supplement: Supporting Information 3 — Table S3: data of all eligible studies included in the review. [file 7891775.f3.docx]

**Table S3:** Data of all eligible studies included in the review

| **Author ID** | **Article title** | **Regional state** | **Study location** | **Collection area** | **Collection methods** | **Total blood fed caught** | **Human blood meal index** | **Bovine blood meal index** | **Mixed blood meal index** | **Unknown blood meal index** | **%** |
| --- | --- | --- | --- | --- | --- | --- | --- | --- | --- | --- | --- |
| Animut et al., 2013 | Blood meal sources and entomological inoculation rates of anopheline along a highland altitudinal transect in south-central Ethiopia | SNNP | South-central Ethiopia, Butajira, (Hobe, Dirama, Wurib) | Both | CDC-LTs | 492 | 218(44.3) | 144(29.3) | 43(8.7) | 87(17.7) | 36.8 |
|  |  |  |  |  | PSC | 841 | 237(28.2) | 248(29.5) | 124(14.7) | 232(27.6) | 62.9 |
|  |  |  |  |  | APS | 3 | 2(66.7) | 1(33.3) | 0.0 | 0.0 | 0.2 |
|  |  |  |  | Total | Three methods | 1,336 | 457(34.2) | 393(29.4) | 167(12.5) | 319(23.9) | 100 |
| Kibret et al., 2014 | Increased malaria transmission around irrigation schemes in Ethiopia and the potential of canal water management for malaria vector control | Oromia | Central Ethiopia (Ziway) | Both | CDC-LTs | 2,335 | 1,649(70.6) | 476(20.4) | 133(5.7) | 77(3.3) | 100 |
| Kibret et al., 2012 | How does Ethiopian dam increase malaria? Entomological determinants around the Koka reservoir | Oromia | South-east Ethiopia (Koka dam) | Both | CDC-LTs | 455 | 264(58.0) | 70(15.4) | 50(11.0) | 71(15.6) | 100 |
| Lulu et al., 1998 | Chromosomal inversion polymorphisms of Anopheles arabiensis from some localities in Ethiopia in relation to host feeding choice | Mixed-regions (SNNP, Gambella & Afar) | South Ethiopia (Sille, Erbore), South west Ethiopia (Jawe), East Ethiopia (Ledi, Alibeti) | Indoor | Mouth aspirator | 538 | 178(33.0) | 138(25.7) | 31(5.8) | 191(35.5) | 100 |
| Hadis et al., 1997 | Host choice by indoor-resting *Anopheles arabiensis* in Ethiopia | Mixed-regions (SNNP, Gambella & Afar) | South Ethiopia (Sille, Erbore), West Ethiopia (Itang, Jawe), East Ethiopia (Ledi, Alibeti) | Indoor | Mouth aspirator | 611 | 144(23.6) | 170(27.8) | 11(1.8) | 286(46.8) | 100 |
| Gari et al., 2016 | Malaria incidence and entomological findings in an area targeted for a cluster-randomized controlled trial to prevent malaria in Ethiopia: results from a pilot study | Oromia | South Ethiopia (Adami-tulu district)) | Both | CDC-LTs | 24 | 15(62.5) | 6(25.0) | 3(12.5) | 0.0 | 26.4 |
|  |  |  |  |  | PSC | 48 | 35(72.9) | 10(20.8) | 3(6.3) | 0.0 | 52.7 |
|  |  |  |  |  | APS | 19 | 4(21.1) | 10(52.6) | 3(15.8) | 2(10.5) | 20.9 |
|  |  |  |  | Total | Three methods | 91 | 54(59.3) | 26(28.6) | 9(9.9) | 2(2.2) | 100 |
| Massebo et al., 2015 | Zoophagic behaviour of anopheline mosquitoes in southwest Ethiopia: opportunity for malaria vector control | SNNP | South-west Ethiopia (Chano) | Both | CDC-LTs | 988 | 94(9.5) | 70(7.1) | 644(65.2) | 180(18.2) | 44.2 |
|  |  |  |  |  | PSC | 352 | 59(16.7) | 154(43.8) | 74(21) | 65(18.5) | 15.6 |
|  |  |  |  |  | APS | 894 | 27(3.0) | 521(58.3) | 89(10.0) | 257(28.7) | 40.0 |
|  |  |  |  | Total | Three methods | 2,234 | 180(8.1) | 745(33.3) | 807(36.1) | 502(22.5) | 100 |
| Zemene et al., 2021 | Malaria vector dynamics and utilization of insecticide-treated nets in low-transmission setting in Southwest Ethiopia: implications for residual transmission | Oromia | South-west Ethiopia (Shebe Sambo district: Kishe) | Both | CDC-LTs | 39 | 20(51.3) | 11(28.2) | 6(15.4) | 2(5.1) | 97.5 |
|  |  |  |  |  | PSC | 1 | 1(100.0) | 0.0 | 0.0 | 0.0 | 2.5 |
|  |  |  |  | Total | Two methods | 40 | 21(52.5) | 11(27.5) | 6(15.0) | 2(5.0) | 100 |
| Assa et al., 2023 | Anopheles mosquito diversity, entomological indicators of malaria transmission and challenges of morphological identification in southwestern Ethiopia | SNNP | South-west Ethiopia (Boreda distrct: Kodo-Awsato-Menuka, Zefine-Menuka) | Indoor | CDC-LTs | 10 | 6(60.0) | 2(20.0) | 0.0 | 2(20.0) | 71.4 |
|  |  |  |  |  | PSC | 4 | 3(75.0) | 0.0 | 0.0 | 1(25.0) | 28.6 |
|  |  |  |  | Total | Two methods | 14 | 9(64.3) | 2(14.3) | 0(0.0) | 3(21.4) | 100 |
| Eba et al., 2021 | Anopheles arabiensis hotspots along intermittent rivers drive malaria dynamics in semi-arid areas of Central Ethiopia | Mixed-regions (Oromia & SNNP) | South-west Ethiopia (Goro district: Rebu; and Abeshege districts: Dire lafto) | Indoor | PSC | 747 | 156(20.9) | 266(35.6) | 81(10.9) | 244(32.6) | 100 |
| Eshetu et al., 2023 | Cattle feeding tendency of Anopheles mosquitoes and their infection rates in Aradum village, North Wollo, Ethiopia: an implication for animal-based malaria control strategies | Amhara | North Ethiopia (Raya Kobo district: Aradum) | Both | Clay pot | 10 | 0.0 | 5(50.0) | 0.0 | 5(50.0) | 23.8 |
|  |  |  |  |  | PSC | 16 | 1(6.2) | 0.0 | 2(12.5) | 13(81.3) | 38.1 |
|  |  |  |  |  | APS | 16 | 2(12.6) | 9(56.2) | 0.0 | 5(31.2) | 38.1 |
|  |  |  |  | Total | Three methods | 42 | 3(7.1) | 14(33.3) | 2(4.8) | 23(54.8) | 100 |
| Kindu et al., 2018 | Study on the species composition and ecology of anophelines in Addis Zemen, South Gondar, Ethiopia | Amhara | North Ethiopia (Libo-Kemkem district: Addis Zemen) | Both | CDC-LTs | 6 | 2(33.3) | 2(33.3) | 0.0 | 2(33.3) | 85.7 |
|  |  |  |  |  | PSC | 0.0 | 0.0 | 0.0 | 0.0 | 0.0 | 0.0 |
|  |  |  |  |  | APS | 1 | 0.0 | 0.0 | 0.0 | 1(100.0) | 14.3 |
|  |  |  |  |  | Clay pot | 0 | 0.0 | 0.0 | 0.0 | 0.0 | 0.0 |
|  |  |  |  | Total | Four methods | 7 | 2(28.6) | 2(28.6) | 0.0 | 3(42.8) | 100 |
| Getachew et al., 2018 | Species composition, blood meal hosts and Plasmodium infection rates of Anopheles mosquitoes in Ghibe River Basin, southwestern Ethiopia | SNNP | South-west Ethiopia (Abeshege district: Wolkite) | Both | CDC-LTs | 218 | 55(25.3) | 92(42.0) | 3(1.5) | 68(31.2) | 33.7 |
|  |  |  |  |  | PSC | 20 | 15(75.0) | 2(10.0) | 1(5.0) | 2(10.0) | 3.1 |
|  |  |  |  |  | APS | 60 | 2(3.2) | 50(83.4) | 1(1.7) | 7(11.7) | 9.3 |
|  |  |  |  |  | HMA | 58 | 43(74.0) | 8(14.0) | 0.0 | 7(12.0) | 9.0 |
|  |  |  |  |  | CMA | 291 | 6(2.0) | 198(68.0) | 2(0.7) | 85(29.3) | 45.0 |
|  |  |  |  | Total | Five methods | 647 | 121(18.7) | 350(54.1) | 7(1.1) | 169(26.1) | 100 |
| Adugna et al., 2021 | Blood meal sources and feeding behavior of anopheline mosquitoes in Bure district, northwestern Ethiopia | Amhara | North-west Ethiopia (Bure district) | Both | CDC-LTs | 208 | 0.0 | 6(2.9) | 191(91.8) | 11(5.3) | 99.5 |
|  |  |  |  |  | PSC | 1 | 0.0 | 0.0 | 0.0 | 1(100) | 0.5 |
|  |  |  |  | Total | Two methods | 209 | 0.0 | 6(2.9) | 191(91.4) | 12(5.7) | 100 |
| Kibret et al., 2017 | Malaria impact of large dams at different eco-epidemiological settings in Ethiopia | Mixed-regions (Afar, Oromia & Amhara) | East-central Ethiopia (Kesem dam), South-east Ethiopia (Koka dam), North-west Ethiopia (Koga dam) | Both | CDC-LTs | 1,818 | 1,164(64.0) | 427(23.5) | 169(9.3) | 58(3.2) | 100 |
| Tirados et al., 2006 | Blood-feeding behaviour of the malarial mosquito Anopheles arabiensis: implications for vector control | SNNP | South Ethiopia (Konso: Fuchucha & Jarso) | Both | PSC | 232 | 96(41.4) | 78(33.6) | 58(25.0) | 0.0 | 23.8 |
|  |  |  |  |  | APS | 742 | 142(19.2) | 276(37.2) | 220(29.6) | 104(14.0) | 76.2 |
|  |  |  |  | Total | Two methods | 974 | 238(24.5) | 354(36.3) | 278(28.5) | 104(10.7) |  |
| Akirso et al., 2023 | High human blood meal index of mosquitoes in Arba Minch town, southwest Ethiopia: an implication for urban vector-borne diseases transmission | SNNP | Southwest Ethiopia (Arba Minch town) | Indoor | CDC-LTs | 140 | 53(37.6) | 1(0.8) | 0.0 | 86(61.6) | 77.8 |
|  |  |  |  |  | Prokopack aspirator | 40 | 37(92.5) | 0.0 | 0.0 | 3(7.5) | 22.2 |
|  |  |  |  | Total | Two methods | 180 | 90(50.0) | 1(0.6) | 0.0 | 89(49.4) |  |
| Tarekegn et al., 2022 | Species composition, monthly distribution and behaviour of adult Anopheles mosquitoes in areas under elimination setting, Dembia district, Northwestern Ethiopia | Amhara | Northwest Ethiopia (Dembia district) | Both | CDC-LTs | 105 | 17(16.4) | 34(32.6) | 4(3.8) | 50(47.2) | 55.6 |
|  |  |  |  |  | PSC | 24 | 2(8.3) | 9(37.5) | 0.0 | 13(54.2) | 12.7 |
|  |  |  |  |  | APS | 41 | 3(7.3) | 17(41.5) | 2(4.9) | 19(46.3) | 21.7 |
|  |  |  |  |  | Mouth aspirator | 19 | 1(5.3) | 8(42.1) | 0.0 | 10(52.6) | 10.0 |
|  |  |  |  | Total | Four methods | 189 | 23(12.2) | 68(36.0) | 6(3.2) | 92(48.6) | 100 |
| Bamou et al., 2021 | Entomological and Anthropological Factors Contributing to Persistent Malaria Transmission in Kenya, Ethiopia, and Cameroon | Oromia | South-west Ethiopia (Bore Tika, Chewaka) | Both | CDC-LTs, | 327 | 4(1.2) | 141(43.1) | 23(7.0) | 159(48.7) | 100.0 |
|  |  |  |  |  | WEC |  |  |  |  |  |  |
|  |  |  |  |  | BA |  |  |  |  |  |  |
|  |  |  |  |  | PSC |  |  |  |  |  |  |
|  |  |  |  |  | HLC |  |  |  |  |  |  |
|  |  |  |  | Total | Five methods | 327 | 4(1.2) | 141(43.1) | 23(7.0) | 159(48.7) | 100.0 |
