# Supplementary material for: Human Blood Index of Anopheles arabiensis in Ethiopia: A Systematic Review and Meta-Analysis
Source: J Trop Med. 2025 Aug 31;2025:7891775. doi: 10.1155/jotm/7891775 (PMC12414622; doi:10.1155/jotm/7891775)
Supplement: Supporting Information 4 — Table S4: quality assessment chart of the included articles. [file 7891775.f4.docx]

**Table S4:** Quality assessment chart of the included articles

| **SN** | **Authors Id** | **JBI critical appraisal checklist** | | | | | | | | | Overall Quality Score (OQS) | **Weight** |
| --- | --- | --- | --- | --- | --- | --- | --- | --- | --- | --- | --- | --- |
|  |  | 1 | 2 | 3 | 4 | 5 | 6 | 7 | 8 | 9 |  |  |
|  | Animut et al., 2013 | + | + | + | + | + | + | + | + | + | 9 | 100 |
|  | Kibret et al., 2014 | - | - | + | + | + | + | + | + | + | 7 | 77.8 |
|  | Kibret et al., 2012 | - | - | + | + | + | + | + | - | + | 6 | 66.7 |
|  | Lulu et al., 1998 | - | - | + | - | + | + | + | - | + | 5 | 55.7 |
|  | Hadis et al., 1997 | - | - | + | - | + | + | + | - | + | 5 | 55.7 |
|  | Gari et al., 2016 | + | + | - | + | + | + | + | + | - | 7 | 77.8 |
|  | Massebo et al., 2015 | + | + | + | + | + | + | + | + | + | 9 | 100 |
|  | Zemene et al., 2021 | - | - | - | + | + | + | + | + | - | 5 | 55.6 |
|  | Assa et al., 2023 | - | + | - | + | + | - | - | + | + | 5 | 55.6 |
|  | Eba et al., 2021 | - | - | + | + | + | + | + | + | + | 7 | 77.8 |
|  | Eshetu et al., 2023 | + | + | - | + | + | + | - | + | - | 6 | 66.7 |
|  | Kindu et al., 2018 | + | + | - | + | + | - | + | - | + | 6 | 66.7 |
|  | Getachew et al., 2018 | + | + | + | + | + | + | + | + | + | 9 | 100 |
|  | Adugna et al., 2021 | + | + | + | + | + | + | + | + | + | 9 | 100 |
|  | Kibret et al., 2017 | + | - | + | + | + | + | + | + | + | 8 | 88.9 |
|  | Tirados et al., 2006 | + | + | + | + | + | + | + | - | + | 8 | 88.9 |
|  | Akirso et al., 2023 | - | - | + | - | + | + | + | + | + | 6 | 66.7 |
|  | Tarekegn et al., 2022 | + | + | + | + | + | + | + | + | + | 9 | 100 |
|  | Bamou et al., 2021 | + | + | + | + | + | - | - | + | + | 7 | 77.8 |
| Overall quality score point | | | | | | | | | | | | 77.8 |

**Key:** + = Yes, - = No
